# BHMPS Inhibits Breast Cancer Migration and Invasion by Disrupting Rab27a-Mediated EGFR and Fibronectin Secretion

**DOI:** 10.3390/cancers14020373

**Published:** 2022-01-12

**Authors:** Jeong-In Park, Kyung-Hee Song, Seong-Mook Kang, Jeeyong Lee, Seong-Jun Cho, Hyun Kyung Choi, Jiyeon Ahn, Jong-Kuk Park, Jaesung Kim, Sang-Gu Hwang, Dae-Seog Lim, Joon Kim, Seung-Youn Jung, Jie-Young Song

**Affiliations:** 1 Division of Radiation Biomedical Research, Korea Institute of Radiological & Medical Sciences, Seoul 01812, Korea; jipark@kirams.re.kr (J.-I.P.); songkh@kirams.re.kr (K.-H.S.); tjdanranr@naver.com (S.-M.K.); jeeyongl@kirams.re.kr (J.L.); ahnjy@kirams.re.kr (J.A.); jkpark@kirams.re.kr (J.-K.P.); jaesung@kirams.re.kr (J.K.); sgh63@kirams.re.kr (S.-G.H.); 2 Laboratory of Biochemistry, Division of Life Sciences, Korea University, Seoul 02841, Korea; joonkim@korea.ac.kr; 3 Low-dose Radiation Research Team, Radiation Health Institute, Korea Hydro & Nuclear Power Co., Ltd., Seoul 01450, Korea; seongjcho@khnp.co.kr; 4 Department of Chemistry, Sogang University, Seoul 04107, Korea; hkchoi45@sogang.ac.kr; 5 Department of Biotechnology, CHA University, Seongnam 13488, Gyeonggi-do, Korea; dslim@cha.ac.kr

**Keywords:** Rab GTPase, invasion, migration, vesicle trafficking, breast cancer

## Abstract

**Simple Summary:**

Numerous studies targeting Rab GTPases and its multiple effectors have been attempted since exocytosis has been shown to alter tumor malignancy by modulating cancer cell behavior and tumor microenvironment. Here, we demonstrated that BHMPS inhibits migration and invasion of breast cancer cells by blocking the interaction between Rab27a and Slp4. BHMPS interfered with vesicle trafficking and secretion by decreasing FAK and JNK activation. In addition, BHMPS suppressed tumor growth in Rab27a-overexpressing MDA-MB-231 xenograft mice. This study highlighted the importance of understanding the mechanisms of Rab27a-mediated metastasis in improving the therapeutic options for metastatic cancers.

**Abstract:**

Our previous work demonstrated that (*E*)-*N*-benzyl-6-(2-(3, 4-dihydroxybenzylidene) hydrazinyl)-*N*-methylpyridine-3-sulfonamide (BHMPS), a novel synthetic inhibitor of Rab27aSlp(s) interaction, suppresses tumor cell invasion and metastasis. Here, we aimed to further investigate the mechanisms of action and biological significance of BHMPS. BHMPS decreased the expression of epithelial-mesenchymal transition transcription factors through inhibition of focal adhesion kinase and c-Jun N-terminal kinase activation, thereby reducing the migration and invasion of breast cancer. Additionally, knockdown of Rab27a inhibited tumor migration, with changes in related signaling molecules, whereas overexpression of Rab27a reversed this phenomenon. BHMPS effectively prevented the interaction of Rab27a and its effector Slp4, which was verified by co-localization, immunoprecipitation, and in situ proximity ligation assays. BHMPS decreased the secretion of epidermal growth factor receptor and fibronectin by interfering with vesicle trafficking, as indicated by increased perinuclear accumulation of CD63-positive vesicles. Moreover, administration of BHMPS suppressed tumor growth in Rab27a-overexpressing MDA-MB-231 xenograft mice. These findings suggest that BHMPS may be a promising candidate for attenuating tumor migration and invasion by blocking Rab27a-mediated exocytosis.

## 1. Introduction

Metastasis is a crucial process in cancer development and is considered a leading cause of cancer therapy failure; however, its control remains a challenge. Primary cancer develops into secondary cancer through metastasis, which includes five key steps: invasion and migration, intravasation, circulation, extravasation, and colonization [1]. Invasion and migration, the first step of metastasis, are commonly referred to as epithelial-mesenchymal transition (EMT) and are considered a major target for cancer therapy. EMT is a biological process by which epithelial cells are transformed into a mesenchymal state and is strongly associated with the development toward an advanced cancer phenotype. During this process, cancer cells release several factors, including growth factors, chemokines, cytokines, and exosomes, to create a tumor microenvironment (TME) favorable for their survival [1,2].

Rab GTPases constitute a large family of small GTPases that regulate membrane identity and vesicle budding, uncoating, motility, and fusion, through complexing with effector proteins such as sorting adaptors, tethering factors, kinases, and phosphatases. These Rab proteins primarily mediate the trafficking of proteins and organelles involved in cell growth, survival, and motility [3,4]. Rab27a is expressed in a subset of specialized secretory cell types and modulates melanosome trafficking in melanocytes by forming a complex with the Rab27a-specific effector melanophilin (Mlph) and the motor protein myosin-Va [5]. Additionally, among the effectors, synaptotagmin-like protein 4 (Slp4) promotes the docking of multivesicular bodies (MVBs) in the plasma membrane by interacting with Rab27a [6]. Furthermore, Rab27a regulates exosome secretion to create a metastatic niche in melanoma and breast cancers [7,8]. Exosomes are spherical, bilayered proteolipids that enclose various molecules, such as proteins, lipids, metabolites, and nucleic acids, which are specifically organized according to the cells and effectively transmit information to the extracellular matrix (ECM) and surrounding cells [9,10]. Particularly, tumor-derived exosomes have been associated with the development of cancer malignancy by promoting cancer cell proliferation, establishing a premetastatic niche, causing ECM degradation, modulating immune cells and stromal cells, and regulating drug resistance [1,9,11]. Altered expression of Rab27 is observed in various human cancers, but the function or mechanism of Rab27 in cancer is not fully understood. Therefore, many researchers have focused on targeting vesicle trafficking and exocytosis pathways because blocking exocytosis may provide novel therapeutic interventions, as it inhibits metastatic cancer and improves cancer therapeutic efficacy [12].

Initially, BMD-11, the BHMPS parent compound, was isolated as a potent protein-protein inhibitor at the binding interface between Rab27a and melanophilin through virtual screening to discover novel melanosome transport inhibitors for functional cosmetics [13]. Since then, we have synthesized 15 BMD-11 derivatives and selected (E)-N-benzyl-6-(2-(3,4-dihydroxy benzylidene)hydrazinyl)-N-methylpyridine-3-sulfonamide (BHMPS), a new Rab27a-targeting synthetic compound that inhibits migration and invasion by downregulating ECM marker levels in A375 melanoma and MDA-MB-231 breast cancer cells [14]. However, the underlying mechanisms remain largely unknown. Therefore, this study further investigated the antimetastatic potential of BHMPS by focusing on Rab27a-mediated signaling pathways.

## 2. Results

### 2.1. BHMPS Suppresses Migration and Invasion of Breast Cancer Cells

**Figure 1:**
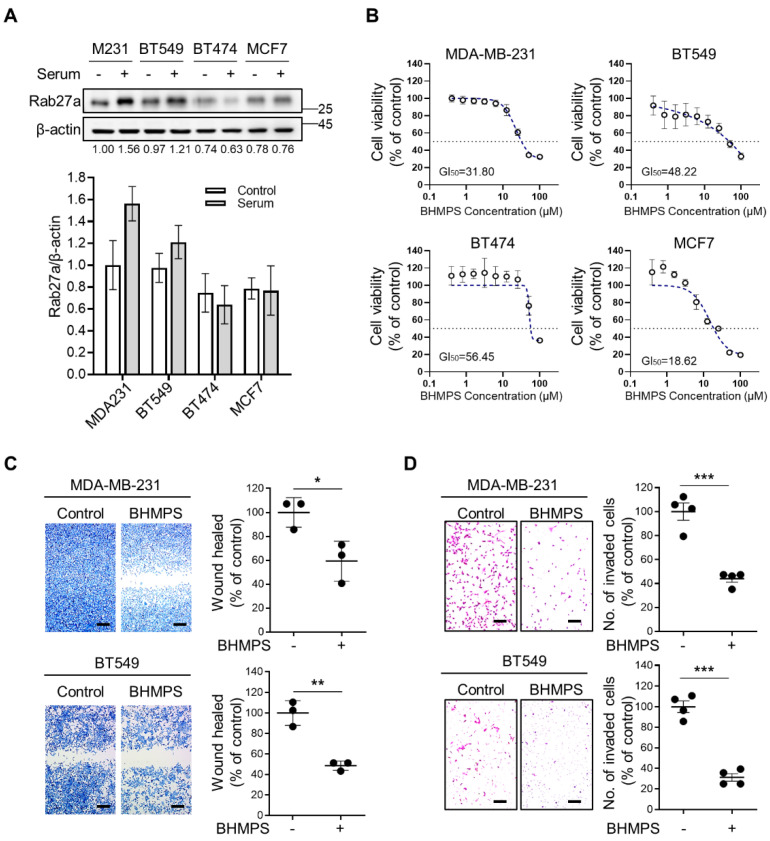
BHMPS inhibits migration and invasion in breast cancer cells. (**A**) Basal levels of Rab27a in several breast cancer cell lines were identified using western blotting. The uncropped images are shown in Supplementary Materials. Relative Rab27a levels were quantified using densitometry with ImageJ software, normalized to β-actin level, and expressed as fold-change compared to that of the MDA-MB-231 control. Data represent the average of three independent experiments. (**B**) MTT assays were performed to determine cell viability. Cells were treated with the indicated concentrations of BHMPS (0–100 µM) for 24 h. Data shown are the means ± SD of three independent experiments. (**C**) MDA-MB-231 and BT549 cells were seeded into 6-well plates and scratched using a 200 µL pipette tip. The cells were treated with 10 µM of BHMPS for 24 h, after which the cells were stained and photographed using a microscope at 40× magnification (Scale bars 250 µm). Percentage wound healing was quantified by measuring the distance of the wound. Data shown are means ± SD of three independent experiments; * *p* < 0.05 and ** *p* < 0.01 compared with the control. (**D**) MDA-MB-231 and BT549 cells were seeded in a Matrigel-coated upper chamber. The lower chamber was treated with 10 µM of BHMPS, and the cells were allowed to invade through a Transwell membrane for 24 h. The cells were stained and photographed using a microscope at a magnification of 40× (Scale bars 250 µm). The invaded cells were counted in five randomly selected regions in each experiment. Data shown are means ± SD of four independent experiments; *** *p* < 0.001 compared with the control.

To confirm the antimetastatic potential of BHMPS, a new synthetic compound that can inhibit Rab27a expression [14], we first verified Rab27a expression in breast cancer cell lines with different subtypes, including MDA-MB-231 and BT549 for TNBC, BT474 for luminal B, and MCF7 for luminal A molecular subtypes. Intrinsic expression of Rab27a protein was higher in MDA-MB-231 and BT549 cells than in BT474 and MCF7 cells (Figure 1A). Moreover, serum stimulation further increased Rab27a expression in the TNBC cells. Next, the effect of BHMPS on cell viability was evaluated using the MTT assay (Figure 1B). MCF7 cells showed the lowest 50% growth inhibition (GI_50_) value of 18.62 µM, whereas BHMPS below 10 µM did not inhibit the growth of other breast cancer cells. Treatment of BHMPS at a non-cytotoxic dose of 10 µM significantly inhibited the migratory and invasive ability of MDA-MB-231 and BT549 cells with high expression of Rab27a (Figure 1C,D).

### 2.2. BHMPS or Rab27a Silencing Decreases Activation of FAK and JNK

**Figure 2:**
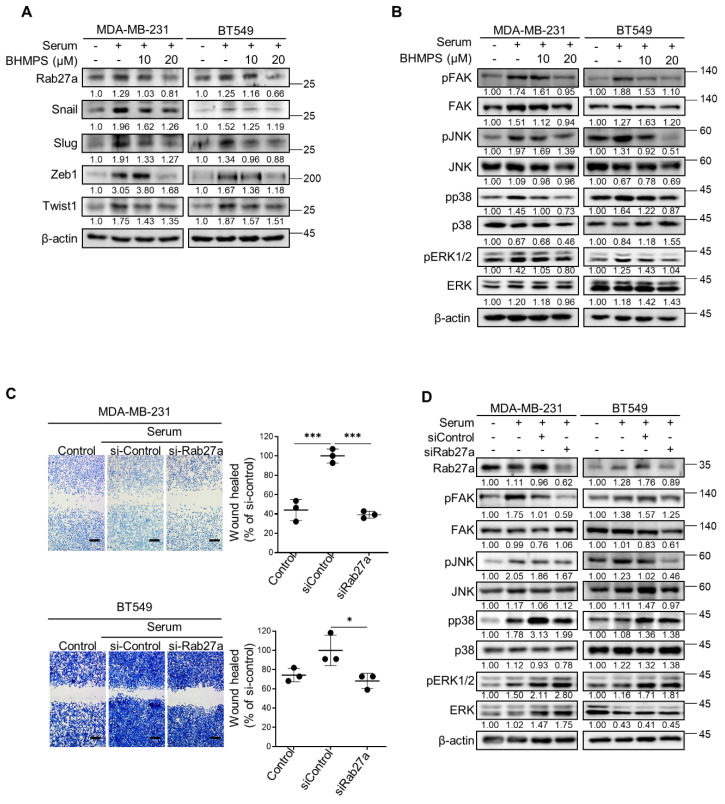
BHMPS inhibits EMT and FAK/MAPK activation. (**A**) Overnight serum-starved cells were stimulated with 10% FBS to induce EMT and co-treated with the indicated concentrations of BHMPS for 24 h. Levels of the specified proteins were compared by western blotting. (**B**) Under the same experimental conditions as (**A**), FAK and MAPK phosphorylation was evaluated by western blotting. (**C**) MDA-MB-231 and BT549 cells were transfected with control siRNA (siControl) or siRab27a. After 24 h, the cells were scratched, and the percentage of migrated cells was determined at 24 h post-scratch. Statistically significant differences are shown in comparison with siControl-transfected cells. Representative images (40 × magnification, Scale bars 250 µm) and means ± SD are from three independent experiments. * *p* < 0.05 and *** *p* < 0.001 compared with siControl-transfected cells. (**D**) Overnight serum-starved cells were stimulated with serum and the indicated target of siRNA for 24 h. FAK and MAPK phosphorylation was determined by western blotting 24 h after transfection. The uncropped images are shown in Supplementary Materials.

To investigate the underlying mechanism of the antimetastatic potential of BHMPS, several signaling molecules involved in invasion and metastasis were examined. BHMPS inhibited the expression of target molecule Rab27a in MDA-MB-231 and BT549 cells (Figure 2A). Additionally, BHMPS effectively decreased the levels of serum-stimulated EMT-activating transcription factors, including Snail, Slug, Zeb1, and Twist1, which are highly related to cancer migration and invasion [15]. Consistent with previous findings that FAK and mitogen-activated protein kinase (MAPK) signaling play essential roles in cancer cell proliferation and metastasis [16,17], FAK, JNK, p38, and ERK1/2 phosphorylation was significantly increased by serum stimulation in both cells, and BHMPS treatment markedly inhibited the phosphorylation of these molecules, especially FAK and JNK (Figure 2B).

When Rab27a expression was suppressed with gene-specific siRNA, the migration of both TNBC cells was inhibited to a similar degree as that of the control without serum stimulation (Figure 2C). Rab27a knockdown also reduced FAK and JNK phosphorylation but did not alter pp38 and pERK1/2 expression in either cell line (Figure 2D).

**Figure 3:**
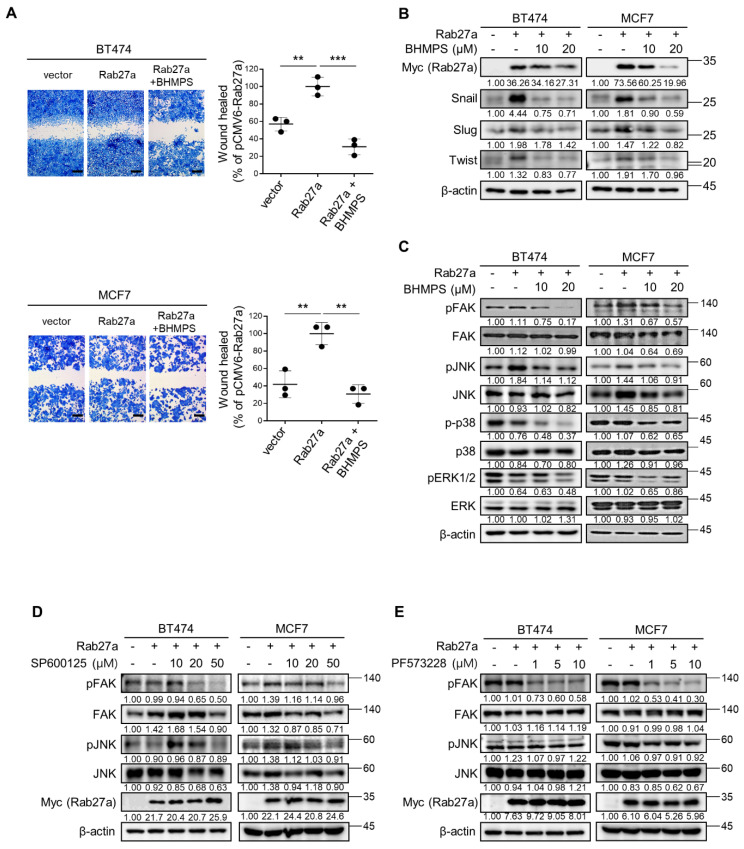
Rab27a overexpression promotes breast cancer cell migration. (**A**) BT474 and MCF7 cells were transfected with control or Rab27a expression vector. After 24 h, the cells were treated with 10 µM of BHMPS, and the percentage of migrated cells was determined. Statistically significant differences are shown in comparison with the Rab27a overexpression group. Representative images (40× magnification, Scale bars 250 µm) and means ± SD are from three independent experiments. ** *p* < 0.01 and *** *p* < 0.001 compared with Rab27a overexpression control. (**B**) BT474 and MCF7 cells were transfected with Rab27a-Myc-DDK expression vector. After 24 h, the cells were treated with the indicated concentrations of BHMPS for 24 h. The immunoblots show the expression of EMT-activating transcription factors. (**C**) Under the same experimental conditions as (**B**), FAK and MAPK signaling was determined by western blotting. (**D**,**E**) After 24 h of Rab27a overexpression, cells were treated with the indicated concentrations of JNK inhibitor (SP600125) or FAK inhibitor (PF573228) for 4 h. The uncropped images are shown in Supplementary Materials.

To further confirm the role of Rab27a and BHMPS in cell migration, Rab27a overexpression was induced in breast cancer cells with low Rab27a expression levels. Rab27a overexpression significantly enhanced cell migration and increased expression of EMT-activating transcription factors in BT474 and MCF7 cells compared with the controls; however, BHMPS treatment effectively suppressed these effects (Figure 3A,B). Additionally, treatment with BHMPS reduced FAK and JNK phosphorylation increased by Rab27a overexpression in both cells (Figure 3C). When Rab27a-overexpressing cells were treated with JNK (SP600125) or FAK (PF573228) inhibitor, SP600125 suppressed FAK phosphorylation but PF573228 did not block JNK phosphorylation, indicating that JNK is an upstream signaling molecule of FAK in Rab27a-overexpressing cells (Figure 3D,E). Taken together, these results suggest that BHMPS suppressed the migration of breast cancer cells by inhibiting Rab27a expression, which transmitted signals to JNK and FAK activation.

### 2.3. BHMPS Inhibits the Interaction of Rab27a with Slp4

**Figure 4:**
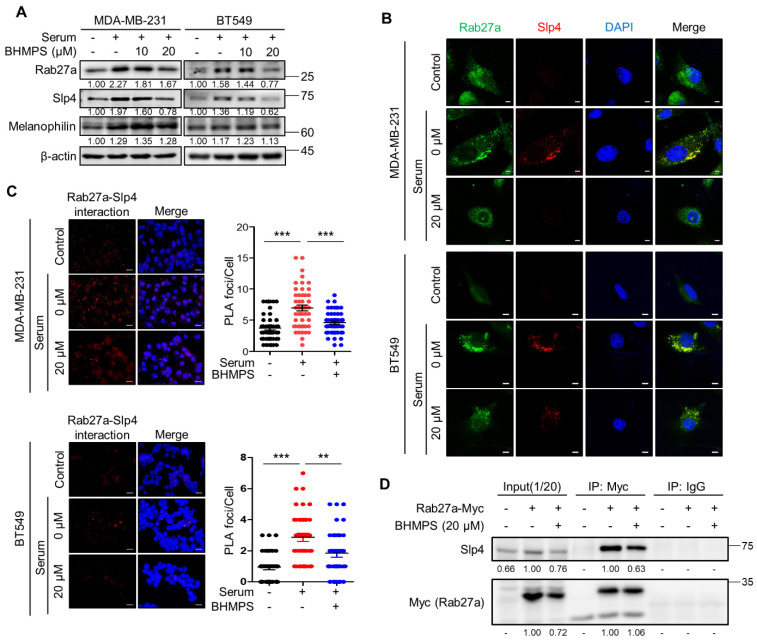
BHMPS disrupts Rab27a and Slp4 interaction. (**A**) Overnight serum-starved cells were stimulated with 10% FBS and co-treated with the indicated concentrations of BHMPS for 24 h. The levels of indicated proteins were measured using western blotting. (**B**) The expression levels of Rab27a (Green) and Slp4 (Red) were detected using specific monoclonal antibodies and an Alexa 488- or 594-conjugated secondary antibody, respectively. Nuclei were stained with DAPI (Blue), and samples were analyzed using confocal microscopy. Representative images from three independent experiments are shown (Scale bars 5 µm). (**C**) Under the same experimental conditions as (**A**), Duolink™ in situ PLA was performed using MDA-MB-231 and BT549 cells. For the assay, both rabbit anti-Rab27a and mouse anti-Slp4 antibodies were used. The interactions (<40 nm) of Rab27a with Slp4 are represented as red dots (Scale bars 20 µm), which were counted in randomly selected regions in each experiment. Nuclei were counterstained with DAPI (Blue). Nuclear foci were counted from at least 50 nuclei. Data are shown as the mean ± SD of the red dots counted per nucleus. ** *p* < 0.01 and *** *p* < 0.001 compared with serum stimulation. (**D**) Rab27a-Myc-DDK-stably expressing MDA-MB-231 cell was produced to verify that BHMPS can inhibit the interaction between Rab27a and Slp4. Protein samples (1000 µg) were immunoprecipitated with a magnetic bead-conjugated Myc-tag mouse monoclonal antibody or mouse normal IgG used as a negative control. Values are expressed as the average intensity of each protein compared to the untreated sample from three independent experiments. The uncropped images are shown in Supplementary Materials.

Rab27a can bind to multiple factors and interact with specific effectors tailored to a particular biological setting [18]. Given that Rab27a interacts with Slp4 and Mlph in breast cancer cells [19], the expression of the two effector molecules was examined. In MDA-MB-231 and BT549 cells, serum stimulation significantly increased the expression of Slp4 and Mlph, along with that of Rab27a. However, BHMPS significantly decreased the expression of Slp4 in a dose-dependent manner compared with that of Mlph (Figure 4A). Therefore, we further examined the interaction between Rab27a and Slp4. As shown in Figure 4B, serum stimulation increased the expression of both Rab27a and Slp4 and their co-localization. Additionally, the number of PLA foci visualized as red fluorescent dots was increased by serum stimulation, indicating that there is an interaction between Rab27a and Slp4. As expected, BHMPS treatment reduced this phenomenon (Figure 4C). To confirm the direct interaction between Rab27a and Slp4, immunoprecipitation was performed using Rab27a-overexpressing MDA-MB-231 cells because the intrinsic levels of Rab27a were insufficient to detect molecular binding. BHMPS treatment decreased the precipitation of Slp4 compared with that of Rab27a-Myc, indicating that BHMPS inhibited the binding of Rab27a and Slp4 in breast cancer cells (Figure 4D).

### 2.4. BHMPS Interferes with Vesicle Trafficking and Secretion

**Figure 5:**
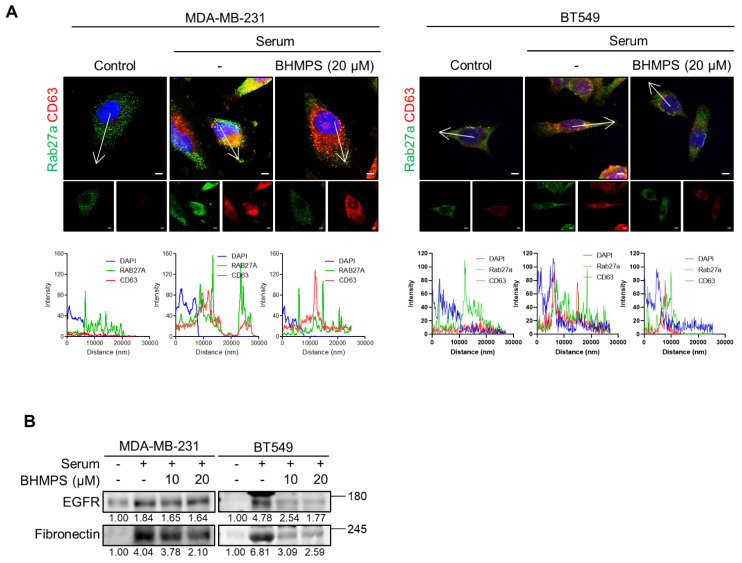
BHMPS interferes with the vesicle secretion process. (**A**) Confocal microscopic analysis was performed to examine the localization of exosome and Rab27a altered by BHMPS in MDA-MB-231 and BT549 cells. Exosome (Red) was labeled with the CD63 antibody and an Alexa 594-conjugated secondary antibody. Rab27a (Green) was labeled with the Rab27a antibody and an Alexa 488-conjugated secondary antibody. DAPI (300 ng/mL) was used to mark the nucleus (Blue). The scale bar represents 5 µm. The single channel images of Rab27a and CD63 are displayed below the left and right of the merged image, respectively. The fluorescence intensities at the marked white arrow were analyzed using Carl Zeiss ZEN lite software and represented in the bottom panel. (**B**) Overnight serum-starved cells were stimulated with 10% FBS and co-treated with the indicated concentrations of BHMPS for 24 h, and then conditioned media were collected. After quantification, levels of the specified proteins were analyzed using western blotting with the same concentration of proteins. The uncropped blots and the images of Ponceau S-stained membrane are shown in Supplementary Materials.

Considering the role of Rab27a in vesicle transport and exocytosis, the intracellular localization of Rab27a and CD63 was examined. A member of the tetraspanin superfamily, CD63, is highly enriched in late endosomal MVBs and exosomes [20]. Serum stimulation induced co-localization of CD63 and Rab27a near the plasma membranes of TNBC cells, while BHMPS accumulated CD63 in the perinuclear region and markedly attenuated this co-localization in the plasma membrane (Figure 5A). These results suggest that Rab27a inhibition by BHMPS could suppress the transport of vesicles to the plasma membrane and secretion. Since EGFR and FN are considered clinically relevant exosome protein markers for breast cancer [21], the expression of EGFR and FN in the conditioned medium was examined. Serum stimulation significantly increased the release of EGFR and FN, and BHMPS effectively suppressed their release (Figure 5B).

### 2.5. BHMPS Inhibits Tumor Growth in Rab27a-Overexpressing Tumor Xenograft Mice

**Figure 6:**
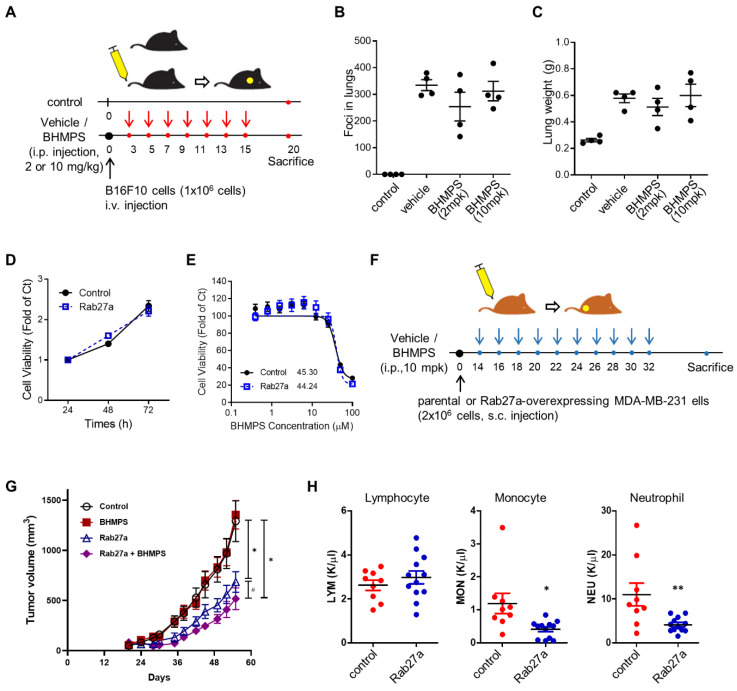
BHMPS suppresses the growth of Rab27a-overexpressed tumor. (**A**) Schematic diagram of in vivo BHMPS treatment in the experimental lung metastasis model. (**B**) B16F10 cells (1 × 10^6^ cells/mouse) were injected intravenously into the tail vein of C57BL/6 mice (*n* = 4 mice/group). Three days after tumor cell injection, BHMPS (2 or 10 mg/kg) or vehicle was administrated intraperitoneally every 2 d for a total of six times. After mouse sacrifice, numbers of cell colonies metastasized to the lungs were counted and the mean ± SE are shown. (**C**) Data shown are the means ± SE of weight of the lungs. (**D**) MTT assay was performed to determine the viability of Rab27a-overexpressing cells. The viability from 24 h to 72 h after seeding with the same number of cells was analyzed. Data shown are the means ± SD of three independent experiments. (**E**) MTT assay was performed to determine the effect of BHMPS on the viability of Rab27a-overexpressing cells. Cells were treated with the indicated concentrations of BHMPS (0–100 µM) for 24 h. Data shown are the means ± SD of three independent experiments. (**F**) Schematic of in vivo BHMPS administration in the tumor xenograft model. (**G**) Control (parental MDA-MB-231 cells) or Rab27a-overexpressed MDA-MB-231 cell (2 × 10^6^ cells/mouse) were injected subcutaneously into the hind thigh of BALB/c nude mice (*n* = 6–8 mice/group). Fourteen days after tumor cell injection, BHMPS (10 mg/kg) or vehicle was administrated intraperitoneally every 2 d for a total of 10 times. The means ± SE of tumor size change over time were shown, and significance was analyzed with repeated measures one-way ANOVA and Tukey’s post hoc comparisons. (**H**) MDA-MB-231 (*n* = 9) or Rab27a-overexpressed MDA-MB-231 cells (*n* = 12) were injected subcutaneously into the hind thigh of BALB/c nude mice. Fifty-five days after tumor cell injection, peripheral blood was collected, and CBC were analyzed. The relative levels of the indicated CBC parameters are shown. Data are shown as the mean ± SE. * *p* < 0.05 and ** *p* < 0.01 compared with control, # *p* < 0.05 compared with Rab27a-overexpressing group.

To investigate whether the above results could be replicated in vivo, the antitumor effect of BHMPS was evaluated in an experimental lung metastasis model of B16F10 melanoma. BHMPS administration did not show any antimetastatic effects (Figure 6A–C). Similarly, tumor growth or metastasis was not affected by BHMPS in the MDA-MD-231 cell xenograft mouse model and the spontaneous metastasis model of 4T1/luc cells (data not shown). Therefore, we further examined tumor growth in Rab27a-overexpressing MDA-MB-231 cell xenograft mice with elevated levels of Rab27a. The viability of Rab27a-overexpressed cells was similar to that of the control parental cells, and the GI_50_ value of BHMPS did not differ between the two cells (Figure 6D,E). Interestingly, tumor growth in mice transplanted with Rab27a-overexpressing cells was significantly delayed compared to that of the parental control cells, and BHMPS was shown to inhibit tumor growth only in Rab27a-overexpressing tumors (Figure 6F,G). To determine whether this phenomenon is related to tumor immunity, a complete blood count (CBC) analysis was performed. The number of monocytes and neutrophils were significantly decreased in Rab27a-overexpressing tumor xenograft mice compared to the control group (Figure 6H). The number of lymphocytes, red blood cells and platelets was not different between the two groups.

### 2.6. Association between Rab27a Expression and OS of Breast Cancer Patients

**Figure 7:**
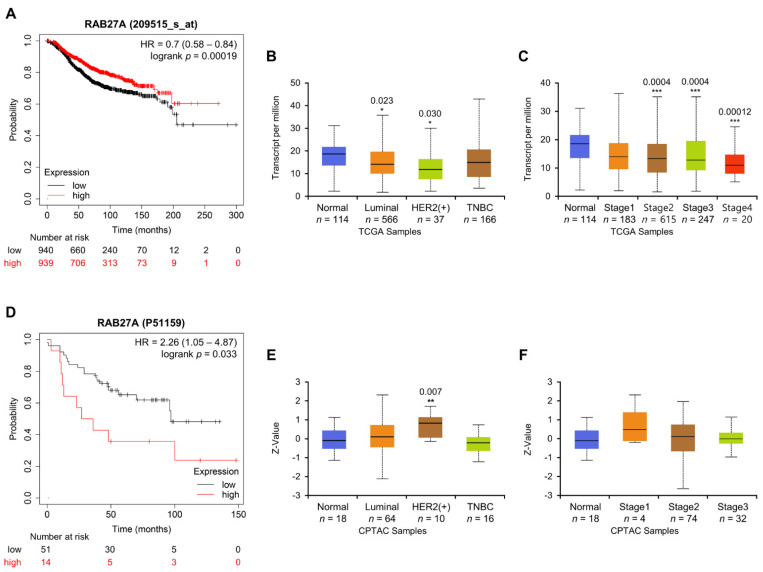
Clinical significance of Rab27a mRNA and protein expression levels. (**A**) Effect of Rab27a mRNA on OS of breast cancer patients, using Kaplan-Meier Plotter analysis. The image with the lowest logrank *p* value is represented. The red and black lines indicate high and low Rab27a expression, respectively. (**B**,**C**) Rab27a mRNA expression levels in (**B**) major subclasses of breast cancer or (**C**) different breast cancer stages were analyzed with the UALCAN tool using TCGA BRCA dataset. (**D**) the overall survival rate of breast cancer patients based on Rab27a expression at the protein level was analyzed using the Kaplan-Meier Plotter. (**E**,**F**) The expression level of Rab27a protein according to (**E**) major subclasses or (**F**) stages of breast cancer was analyzed with UALCAN tool using the CPTAC breast cancer dataset. Boxplot represents median with upper and lower quartiles, and the bars represent the maximum and minimum values. Compared with the normal tissue control group, statistically significant differences were indicated as * *p* < 0.05, ** *p* < 0.01, and *** *p* < 0.001.

Given the conflicting results of Rab27a overexpression in in vitro and in vivo studies, we analyzed the clinical significance of Rab27a expression levels using public expression profiles. Rab27a expression was significantly higher in glioblastoma and thyroid carcinoma than in adjacent normal tissues but lower in breast cancer (Supplementary Figure S1). Investigating the association between Rab27a expression and patient survival showed that OS in breast cancer patients was longer in the high Rab27a expression group than that in the low Rab27a expression group (Figure 7A). Additionally, Rab27a expression was significantly lower in the luminal or Her-2 positive subclasses as well as in stage 2 to 4 invasive breast carcinoma than in the normal group (Figure 7B,C). In contrast to the above observations, patients with high levels of Rab27a protein showed poor survival, and there was no difference in the expression of Rab27a protein between tumor stages. The expression of Rab27a was significantly higher only in the Her-2 positive subgroup than in the normal group (Figure 7D–F). The value of Rab27a as a prognostic marker in cancer patients was assessed differentially by dataset (sample size), cancer type, and analysis program. According to the Human Protein Atlas, high expression of Rab27a decreased the survival rate of renal cancer patients but increased that of breast cancer patients. Therefore, it remains controversial whether high Rab27a expression is suitable for predicting cancer prognosis.

## 3. Discussion

We demonstrated that BHMPS could effectively inhibit the migration potential of breast cancer cells with high expression of Rab27a through down-regulation of FAK and JNK signaling pathways. FAK is a non-receptor tyrosine kinase that mediates signal transduction by integrins and other cell surface receptors to regulate cell adhesion, migration, survival, differentiation, and metastasis in a variety of cells [22,23]. FAK is known as a crucial downstream signaling molecule in the trafficking of integrin that continuously undergoes endo/exocytic trafficking to facilitate focal adhesion turnover, cell migration, invasion, and cytokinesis, resulting in altered polymerization or stabilization of actin and microtubule filaments [24,25]. FAK aggravates cancer progression by activating the Src, PI3K-Akt, and Raf-MEK-ERK signaling pathways [26], and the deletion of FAK in mammary epithelial cells suppresses tumor formation and progression in mouse models of breast cancer [27,28,29]. JNKs are MAPK family enzymes that are best known for regulating proapoptotic signaling and transcriptional activation, but they are also involved in the expression of EMT markers and matrix metalloproteinases via the regulation of c-Jun and AP-1, which stimulate cell motility [30,31,32]. In line with these observations, BHMPS treatment or silencing of Rab27a significantly decreased FAK and JNK activation, with a reduction in the levels of EMT-activating transcription factors.

Rab27a has been reported to regulate vesicle transport and exocytosis by acting as a compartment-specific molecular switch [4,33]. Rab27 consists of two isoforms, Rab27a and Rab27b, which share high sequence similarity (71% amino acid identity) but function differently even in the same cell type [34,35]. For example, knockdown of Rab27a (or its potential effector Slp4) causes a decrease in the number of plasma membrane-docked multivesicular endosomes (MVEs) and increased sizes of MVEs, whereas knockdown of Rab27b (or Slac2-b) causes perinuclear clustering of MVEs and a decrease in the size of the MVEs in HeLa cells [36]. Moreover, only Rab27a, not Rab27b, is required for exosome secretion by murine mammary carcinoma cells [37]. Several studies have shown that Rab27a plays a crucial role in exosome secretion, such as insulin secretion by pancreatic β-cells, melanosome transport by melanocytes, cytotoxic granule exocytosis by immune cells, and reactive oxygen species (ROS) production by macrophages and neutrophils [6,35,37,38]. Although the contents of exosomes were not identified owing to experimental limitations in this study, the inhibition of Rab27a expression significantly decreased the secretion of oncogenic EGFR and the ECM protein FN. Because EGFR is frequently overexpressed in TNBC than in normal tissue and contributes to malignancy and invasiveness [39,40], Rab27a may be a promising target to disrupt these secretory pathways. However, since there are more than 65 Rab protein subfamilies and several factors related to Rab proteins, including effectors, binding proteins, and regulators, further studies are required to determine whether the interaction between Slp4 and Rab27a has sufficient specificity and selectivity to be responsible for Rab27a-mediated vesicle biogenesis and trafficking.

Several studies have demonstrated that Rab27a blockade decreased primary tumor growth and the number of metastatic lung foci in 4T1 cell-transplanted mice but not in TS/A cell-injected mice [12,37]. Additionally, upregulation of Rab27a expression promotes rapid growth of primary tumors and increases metastatic potential [7,12]. However, we found that Rab27a-overexpressing MDA-MB-231 xenograft tumors grew significantly slower than control tumors, and the anticancer activity of BHMPS was only observed in Rab27a-overexpressing tumor xenografts. A recent study reported that ionizing radiation accelerates the exosome secretion pathway by upregulating Rab27a and Rab27b levels in MCF-7 cells, thereby increasing ROS production and apoptosis [41]. This finding suggests the possibility that Rab27a-mediated MVE secretion may have a detrimental effect not only on the secretory cells themselves but also on adjacent cells of the TME. Moreover, since accumulation of neutrophils in tumor-bearing mice modifies TME by secretion of factors that promote angiogenesis and increase tumor cell migration [33,42,43], the significant decrease of neutrophils in Rab27a-overexpressing tumor-bearing mice in this study may be another plausible explanation for the unexpected growth inhibition of Rab27a-overexpressing tumors. It has been documented that neutrophils are the predominant circulating leukocyte population in humans and tumor-associated neutrophils and their myeloid precursors in the spleen, bone marrow, and blood play an important role in cancer biology [44]. Furthermore, neutrophil infiltration has been associated with poor prognosis in some cancer patients, but further investigation into the polarization, conversion, and function regulation of N1 or N2 is required. We speculate that the significant decrease in neutrophils in Rab27a-overexpressing tumor-bearing mice may be attributed to the secretory substances that reduce the number of neutrophils or inhibit neutrophil attraction. Secreted molecules may vary depending on stimuli, the binding partners of Rab27a, specific cell types, etc. For example, endogenous Rab27a may produce tumor-promoting factors, while excess Rab27a may additionally release more toxic and cytocidal factors. Future studies should be directed towards examining these possibilities to further dissect the mechanism involved.

In contrast to the observation that Rab27a expression was upregulated in clinical cancer patients [45,46], our analysis using public databases showed different correlations between mRNA and protein levels. There is a considerably greater shortage of publicly available proteome databases than transcriptome databases such as TCGA, and using datasets with different data often produces different results. Interestingly, it has been reported that the evident discrepancies between transcript and protein levels, such as groups with high protein levels but low transcript levels, were mostly associated with vesicle-mediated transport. The negative correlation may be explained by protein transport and spatial separation [47]. According to a recent meta-analysis evaluating the prognostic significance of Rab27 expression in solid tumors, poor survival was significantly associated with high Rab27b expression but not with high Rab27a expression [48]. Moreover, Shi et al. reported that high expression of Rab27a indicates a favorable prognosis, contrary to other studies [49]. Therefore, more thorough validation and a careful approach will be required to apply Rab27a as a promising prognostic marker and as a druggable target.

There are some limitations in the present study that should be addressed. First, the involvement of other Rab27 families in breast cancer invasion and metastasis remains to be elucidated, particularly with regard to how cells select Rab27a among approximately 70 Rab families. Second, it is necessary to determine the contents loaded into exosomes, which can expand the application of Rab27a as a valuable biomarker. Accordingly, the binding partners of Rab27a need to be identified, and whether they are changeable depending on the environment also needs to be determined. Third, it should be validated whether Rab27a is a critical factor in breast cancer progression and/or metastasis. In this case, the similarities and differences of Rab27a-mediated exocytosis between immune cells and in cancer should be established. Lastly, further investigation is needed to determine whether off-target effects of BHMPS exist. These will advance our fundamental understanding of Rab27a-mediated exocytosis and metastasis.

## 4. Materials and Methods

### 4.1. Materials

Antibodies and chemicals are listed in the Supplementary Table S1 for vendor and catalog numbers. BHMPS (C_20_H_20_N_4_O_4_S, MW: 412.46) was synthesized using a previously reported method [14] or purchased from 4chem laboratory (Suwon, Gyeonggi-do, Korea).

### 4.2. Cell Cultures

Human breast cancer cells, MDA-MB-231, BT549, BT474, and MCF7 were obtained from the American Type Culture Collection (ATCC, Manassas, VA, USA). BT549 cells were cultured in RPMI 1640 supplemented with 10% FBS, penicillin (100 units/mL), and streptomycin (100 µg/mL). MDA-MB-231 and BT474 cells were grown in DMEM supplemented with 10% FBS, penicillin (100 units/mL), and streptomycin (100 µg/mL). MCF7 cells were maintained in MEM supplemented with 10% FBS, penicillin (100 units/mL), and streptomycin (100 μg/mL). Cells were incubated in a humidified incubator at 37 °C in a 5% CO_2_ atmosphere.

### 4.3. Cell Viability Assay

Cancer cells were seeded in 96-well plates (1 × 10^4^ cells/well). The cells were treated with two-fold serial dilutions of BHMPS from 100 µM to 0.4 µM for 24 h and further incubated with MTT dye solution (0.5 mg/mL) for 4 h. The formed formazan crystals were dissolved in DMSO, and the absorbance was measured using a microplate reader at 550 nm.

### 4.4. Migration Assay

Cells were seeded in 6-well plates (5 × 10^5^ cells/well). After 24 h, the cells were scratched using a 200 μL pipette tip, followed by the administration of 10 µM BHMPS. The migrated cells were stained with 0.05% crystal violet, photographed at 40× magnification, and analyzed under a microscope (Olympus IX73; Olympus, Tokyo, Japan). The data taken were the distance between the two migrated surfaces of cells coming from either side of the wounded area.

### 4.5. Invasion Assay

For the Matrigel invasion assay, cells (5 × 10^4^ cells/well) in serum-free medium were loaded into the upper well of Matrigel-coated Transwell chamber with 8 µm pore size (Corning Inc., Corning, NY, USA), while the lower well was filled with culture medium. After 24 h, the cells that migrated to the lower surface of the membrane were counted in 10 microscopic fields per well, and the average value was quantified.

### 4.6. Western Blot Analysis

Cells were lysed using buffer containing 50 mM Tris-HCl (pH 7.4), 1% NP-40, 150 mM NaCl, 1 mM EDTA, 1 mM PMSF, 1 µg/mL aprotinin, 1 mM Na_3_VO_4_, and 1 mM NaF. Proteins were separated using SDS-PAGE and transferred onto nitrocellulose membranes (GE Healthcare Life Science, Little Chalfont, UK). Proteins were analyzed using the specified antibodies and an ECL detection system (GE Healthcare). ImageJ software version 1.53 h (National Institutes of Health, Bethesda, MD, USA) was used for analysis. Representative images from three or more experiments are shown.

### 4.7. Transfection

Cells were transfected with plasmids and/or small interfering RNA (siRNA) (20 nM) using Lipofectamine 2000 or Lipofectamine RNAiMAX (Invitrogen, Carlsbad, CA, USA) according to the manufacturer’s protocol. For the establishment of stable cell, MDA-MB-231 cells were transfected with pCMV6-Rab27a-Myc-DDK expression vector (#RC217900; OriGene, Rockville, MD, USA) and supplied with 1000 µg/mL of G418 (#ant-gn-1; InvivoGen, San Diego, CA, USA)-containing media. Visible individual colonies were transferred into multi-well plates, after which stably expressing cells were selected using qPCR and western blot analysis. A B6 clone was finally obtained.

### 4.8. Immunoprecipitation

Cell lysates were used for immunoprecipitation with magnetic bead-conjugated Myc-Tag antibodies according to the manufacturer’s protocol. Immunoprecipitated proteins were subjected to SDS-PAGE using the specified antibody. Negative-control experiments were performed in the same way using normal IgG and magnetic beads with the same amount of protein.

### 4.9. Immunofluorescence Confocal Microscopy

Paraformaldehyde-fixed cells were incubated with the indicated primary and fluorescent dye-conjugated secondary antibodies (Invitrogen). Slides were mounted using Duolink Mounting Media with DAPI and images were acquired under an LSM880 confocal microscope (Zeiss, Jena, Germany) using the Zen 2.3 software (Zeiss) for image processing and analysis. Representative images are shown from three or more independent experiments.

### 4.10. Proximity Ligation Assay (PLA)

Direct protein–protein interactions were visualized using in situ PLA [50] using Duolink In Situ Red Starter Kit Mouse/Rabbit (Sigma-Aldrich). After 24 h incubation of cells treated with serum and/or BHMPS, the cells were fixed, permeabilized, blocked, and co-incubated with anti-Rab27a and anti-Slp4 primary antibodies overnight. The next day, Duolink anti-rabbit PLUS and anti-mouse MINUS secondary antibodies and the red detection reagent were treated. After amplification of PLA signals, images were acquired under an LSM880 confocal microscope (Zeiss) and were analyzed with the Zen 2.3 software (Zeiss).

### 4.11. Tumor Xenograft Mouse Model

Six-week-old female C57BL/6 and athymic BALB/c nude mice (Orient Bio Inc., Seongnam, Korea) were housed under specific pathogen-free conditions in microisolator cages and supplied laboratory chow and water *ad libitum*. In the experimental lung metastasis model, B16F10 cells (1 × 10^6^ cells/100 μL phosphate-buffered saline (PBS)) were injected intravenously into the tail vein of C57BL/6 mice, and BHMPS was administered six times every 2 d starting from the 3rd day. The mice were sacrificed, and the lungs were harvested after 20 d of tumor cell injection. In the xenograft model, MDA-MB-231 cells and MDA-MB-231 cells stably overexpressing Rab27a (2 × 10^6^ cells) in 0.1 mL PBS with 50% Matrigel were injected subcutaneously into the thigh hind leg of BALB/c nude mice. Tumors were measured along two axes (L, longest axis; W, shortest axis) with a Vernier caliper (Mitutoyo, Kawasaki, Japan) two or three times a week, and the tumor volume was calculated as [(L × W^2^)/2 (mm^3^)]. When the tumor volumes reached approximately 150 mm^3^, the mice were treated with BHMPS (10 mg/kg of bodyweight) every 2 d for a total of 10 times. These experiments were reviewed and approved by the Institutional Animal Care and Use Committee of the Korea Institute of Radiological and Medical Sciences (KIRAMS 2020-0036).

### 4.12. Bioinformatics Analysis

To analyze the clinical significance of Rab27a expression levels, The Cancer Genome Atlas (TCGA) data were investigated using the TIMER2.0 database (http://timer.cistrome.org/) (accessed on 2 July 2021). The overall survival (OS) according to Rab27a mRNA and protein expression in breast cancer were analyzed using the Kaplan-Meier Plotter (https://kmplot.com/analysis/) (accessed on 18 June 2021). The patient group was split using the median value, and the result of the dataset with the lowest *p*-value for each analysis was represented. The UALCAN website (http://ualcan.path.uab.edu/) (accessed on 5 July 2021) was used to analyze Rab27a expression in major subclasses and at different stages of breast cancer at the mRNA and protein levels.

### 4.13. Data Analysis

Data were presented as mean ± standard deviation (SD) except for the mouse model experiments expressed as mean ± standard error (SE). Statistical differences between groups were analyzed by Student’s *t*-test (two-tailed) or analysis of variance (ANOVA) using GraphPad Prism software version 9.0 (GraphPad, La Jolla, CA, USA). *p*-values lower than 0.05 were considered statistically significant.

## 5. Conclusions

In conclusion, our findings demonstrated for the first time that BHMPS regulates the secretory process of intracellular vesicles by disrupting the interaction between Rab27a and Slp4 in breast cancer cells. FAK and JNK activation regulate Rab27a-mediated exocytosis for the secretion of EGFR and FN, which facilitate migration and invasion. This study also highlighted the importance of understanding the mechanisms of Rab27a-mediated metastasis, which are still largely unknown, in improving the therapeutic options for metastatic cancers.

## Data Availability

All other relevant data are available from the corresponding author upon reasonable request.

## Supplementary Materials

The following supporting information can be downloaded at: https://www.mdpi.com/article/10.3390/cancers14020373/s1, Figure S1: The expression of Rab27a in human tumor and adjacent normal tissues; Table S1: List of details for the antibodies and chemicals. File S1: Full Western blot images.

## Abbreviation

BHMPS, (*E*)-*N*-benzyl-6-(2-(3, 4-dihydroxybenzylidene) hydrazinyl)-*N*-methylpyridine-3-sulfonamide; EMT, epithelial-mesenchymal transition; EGFR, epidermal growth factor receptor; FAK, focal adhesion kinase; JNK, c-Jun N-terminal kinase; siRNA, small interfering RNA; Slp, synaptotagmin-like protein; PLA, proximity ligation assay.
