# Supplementary material for: BHMPS Inhibits Breast Cancer Migration and Invasion by Disrupting Rab27a-Mediated EGFR and Fibronectin Secretion
Source: Cancers (Basel). 2022 Jan 12;14(2):373. doi: 10.3390/cancers14020373 (PMC8773646; doi:10.3390/cancers14020373)
Supplement: Supplementary file 1 [file cancers-14-00373-s001.zip › cancers-1476801-supplementary.pdf]

cancers

MDPI

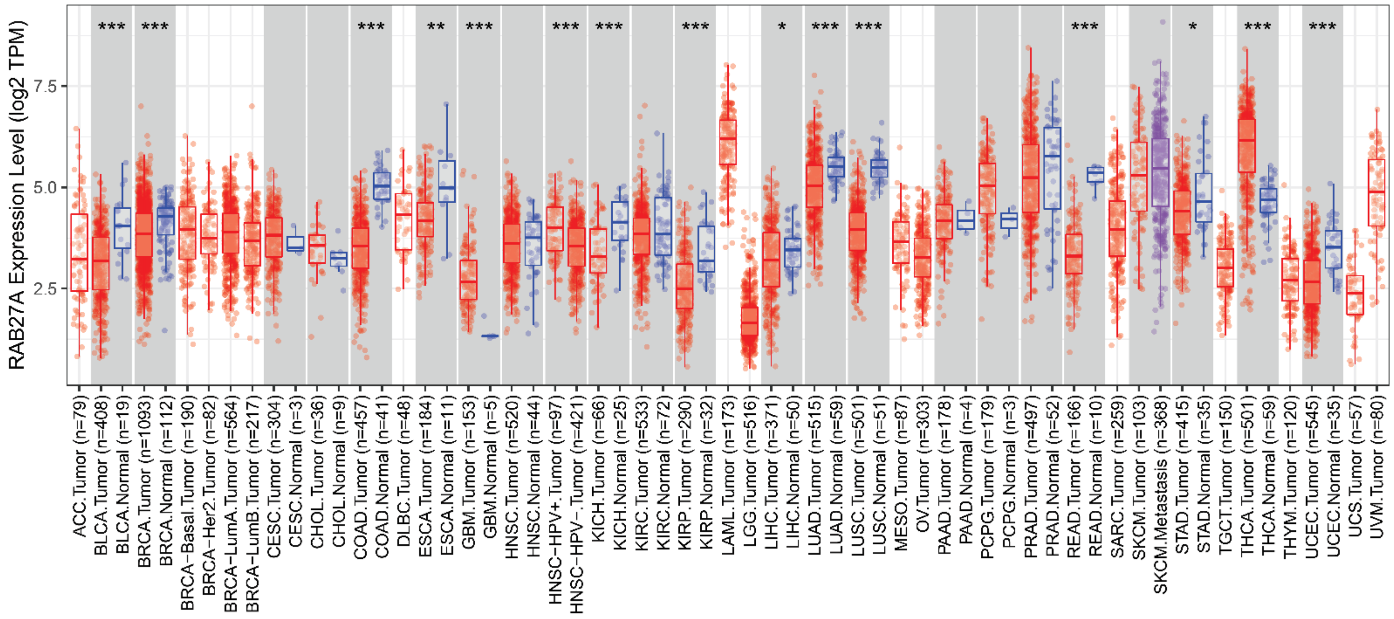

Figure S1. The expressions of Rab27a in human tumors and adjacent normal tissues. The differential expression of Rab27a between tumor and adjacent normal tissues was investigated in all TCGA tumors using Gene_DE module of TIMER2.0 (http://timer.comp-genomics.org/). The distribution of Rab27A expression levels is shown using box plots. Statistical significance is annotated with the number of stars calculated by the Wilcoxon test (*: *p*-value <0.05; **: *p*-value <0.01; ***: *p*-value <0.001). If there is comparable normal tissue data exist, it is displayed as gray columns.

Table S1. List of details for the antibodies and chemicals.

material	vendor	catalog#
Minimal essential medium (MEM)	Welgene (Gyeongsangbuk-do, Korea)	#LM007-07
Dulbecco's modified Eagle's medium (DMEM)	Welgene (Gyeongsangbuk-do, Korea)	#LM001-05
RPMI 1640 medium	Welgene (Gyeongsangbuk-do, Korea)	#LM011-01
fetal bovine serum (FBS)	Welgene (Gyeongsangbuk-do, Korea)	#S001-03
penicillin-streptomycin solution	Welgene (Gyeongsangbuk-do, Korea)	#LS202-02
Dulbecco's phosphate-buffered saline	Welgene (Gyeongsangbuk-do, Korea)	#LB001-02
Matrigel	BD Biosciences (San Jose, CA, USA)	#354234
Rab27a	Cell Signaling Technology (Beverly, MA, USA)	#69295
snail family transcriptional repressor 1 (SNAI1/Snail)	Cell Signaling Technology (Beverly, MA, USA)	#3789
snail family transcriptional repressor 2 (SNAI2/Slug)	Cell Signaling Technology (Beverly, MA, USA)	#9585
zinc finger E-box-binding homeobox 1 (Zeb1)	Cell Signaling Technology (Beverly, MA, USA)	#3396
focal adhesion kinase (FAK)	Cell Signaling Technology (Beverly, MA, USA)	#13009
pFAK (Y397)	Cell Signaling Technology (Beverly, MA, USA)	#8556
c-Jun N-terminal kinases (JNK)	Cell Signaling Technology (Beverly, MA, USA)	#9252
pJNK (T183/Y185)	Cell Signaling Technology (Beverly, MA, USA)	#9255
p38	Cell Signaling Technology (Beverly, MA, USA)	#9212
pp38 (T180/Y182)	Cell Signaling Technology (Beverly, MA, USA)	#4511
ERK	Cell Signaling Technology (Beverly, MA, USA)	#4659
pERK1/2 (T202/Y204)	Cell Signaling Technology (Beverly, MA, USA)	#4370
Myc-tag	Cell Signaling Technology (Beverly, MA, USA)	#2278
epidermal growth factor receptor (EGFR)	Cell Signaling Technology (Beverly, MA, USA)	#4267
synaptotagmin-like protein 4 (Slp4)	Santa Cruz Biotechnology (Dallas, TX, USA)	#sc-374544
fibronectin (FN)	Santa Cruz Biotechnology (Dallas, TX, USA)	#sc-8422
twist family BHLH transcription factor 1(Twist1)	Abcam (Cambridge, MA, USA)	#ab50887
melanophilin (Mlph)	Abcam (Cambridge, MA, USA)	#ab112954

β-actin	Sigma-Aldrich (St. Louis, MO, USA)	#A5316
cell lysis buffer (10×)	Cell Signaling Technology (Beverly, MA, USA)	#9803
Myc-Tag (71D10) rabbit monoclonal antibody	Cell Signaling Technology (Beverly, MA, USA)	#2278
magnetic bead-conjugated Myc-Tag (9B11) mouse monoclonal antibody	Cell Signaling Technology (Beverly, MA, USA)	#5698
horseradish peroxidase (HRP)-conjugated anti-mouse IgG light chain-specific (D3V2A) antibody	Cell Signaling Technology (Beverly, MA, USA)	58802
HRP-conjugated anti-rabbit IgG antibody	Cell Signaling Technology (Beverly, MA, USA)	#7074
3× loading buffer	Cell Signaling Technology (Beverly, MA, USA)	#7722
rabbit Rab27a	Cell Signaling Technology (Beverly, MA, USA)	#69295
rabbit pFAK	Cell Signaling Technology (Beverly, MA, USA)	#8556
rabbit Paxillin	Cell Signaling Technology (Beverly, MA, USA)	#12065
Mouse Rab27a	Santa Cruz Biotechnology (Dallas, TX, USA)	#sc-136996
mouse Slp4	Santa Cruz Biotechnology (Dallas, TX, USA)	#sc-374544
rabbit CD63	Santa Cruz Biotechnology (Dallas, TX, USA)	#sc-15363
