# Supplementary material for: BHMPS Inhibits Breast Cancer Migration and Invasion by Disrupting Rab27a-Mediated EGFR and Fibronectin Secretion
Source: Cancers (Basel). 2022 Jan 12;14(2):373. doi: 10.3390/cancers14020373 (PMC8773646; doi:10.3390/cancers14020373)
Supplement: Supplementary file 1 [file cancers-14-00373-s001.zip › File S1.pdf]

Figure 1-A

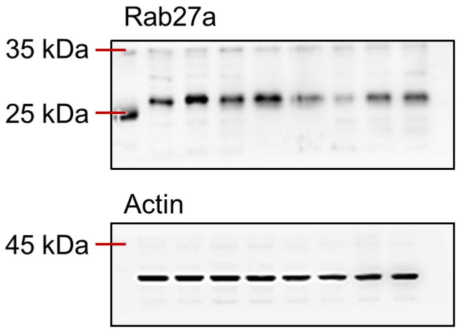

Figure 2-A

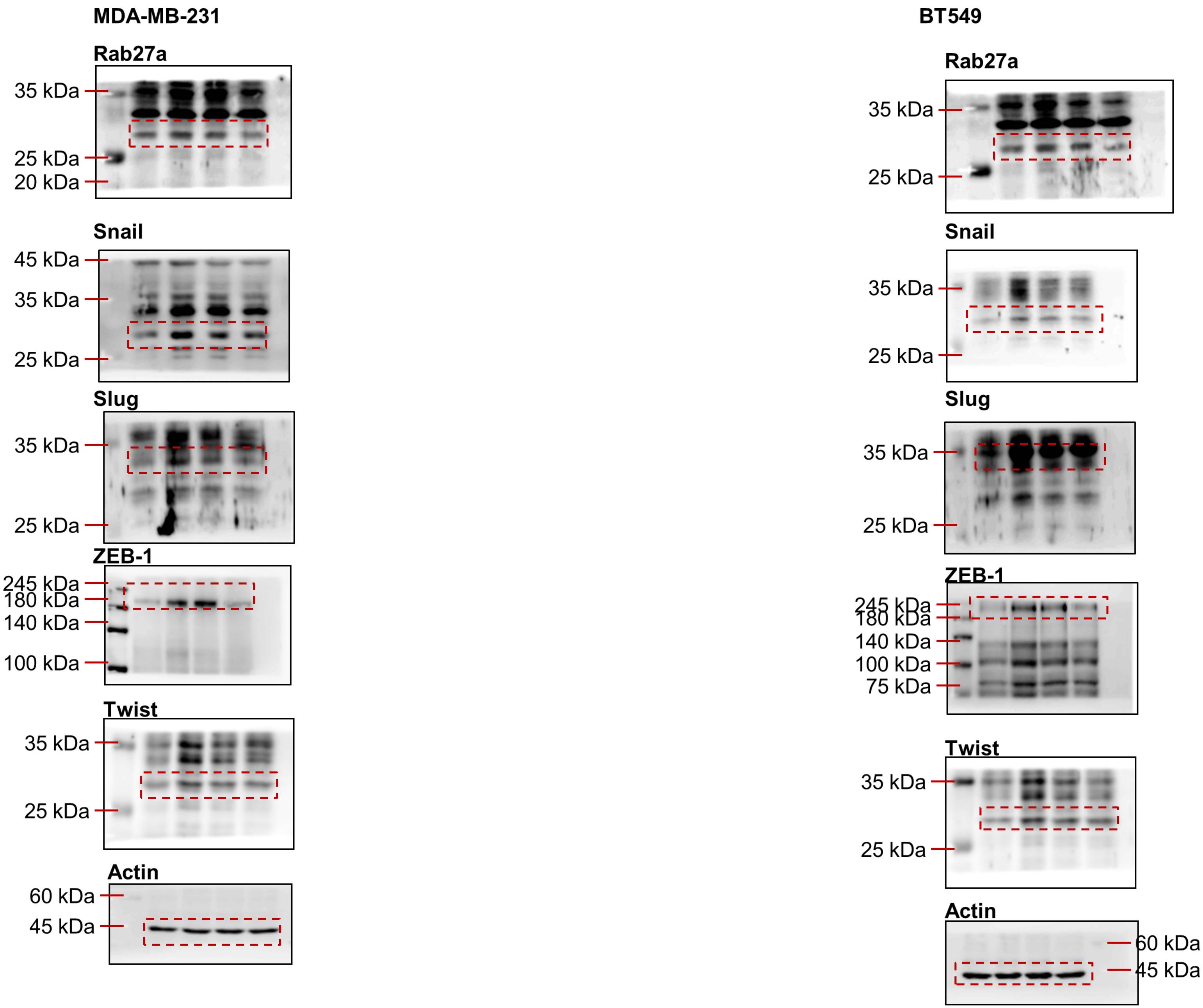
MDA-MB-231
Rab27a
35 kDa
25 kDa
20 kDa
Snail
45 kDa
35 kDa
25 kDa
Slug
35 kDa
25 kDa
ZEB-1
245 kDa
180 kDa
140 kDa
100 kDa
Twist
35 kDa
25 kDa
Actin
60 kDa
45 kDa
BT549
Rab27a
35 kDa
25 kDa
Snail
35 kDa
25 kDa
Slug
35 kDa
25 kDa
ZEB-1
245 kDa
180 kDa
140 kDa
100 kDa
75 kDa
Twist
35 kDa
25 kDa
Actin
60 kDa
45 kDa

Figure 2-B

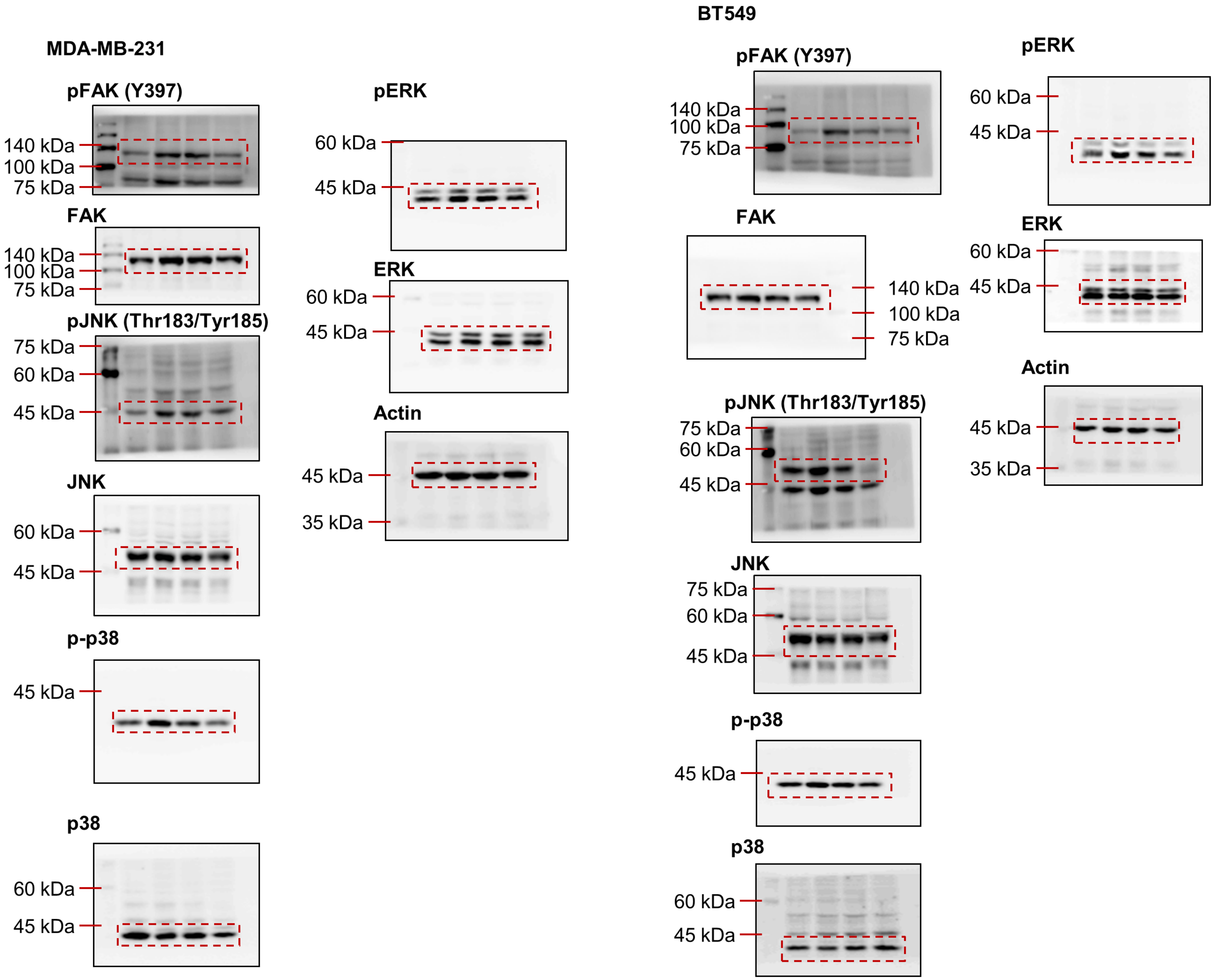
MDA-MB-231
pFAK (Y397)
140 kDa
100 kDa
75 kDa
FAK
140 kDa
100 kDa
75 kDa
pJNK (Thr183/Tyr185)
75 kDa
60 kDa
45 kDa
JNK
60 kDa
45 kDa
p-p38
45 kDa
p38
60 kDa
45 kDa
pERK
60 kDa
45 kDa
ERK
60 kDa
45 kDa
Actin
45 kDa
35 kDa
BT549
pFAK (Y397)
140 kDa
100 kDa
75 kDa
FAK
140 kDa
100 kDa
75 kDa
pJNK (Thr183/Tyr185)
75 kDa
60 kDa
45 kDa
JNK
75 kDa
60 kDa
45 kDa
p-p38
45 kDa
p38
60 kDa
45 kDa
pERK
60 kDa
45 kDa
ERK
60 kDa
45 kDa
Actin
45 kDa
35 kDa

Figure 2-D

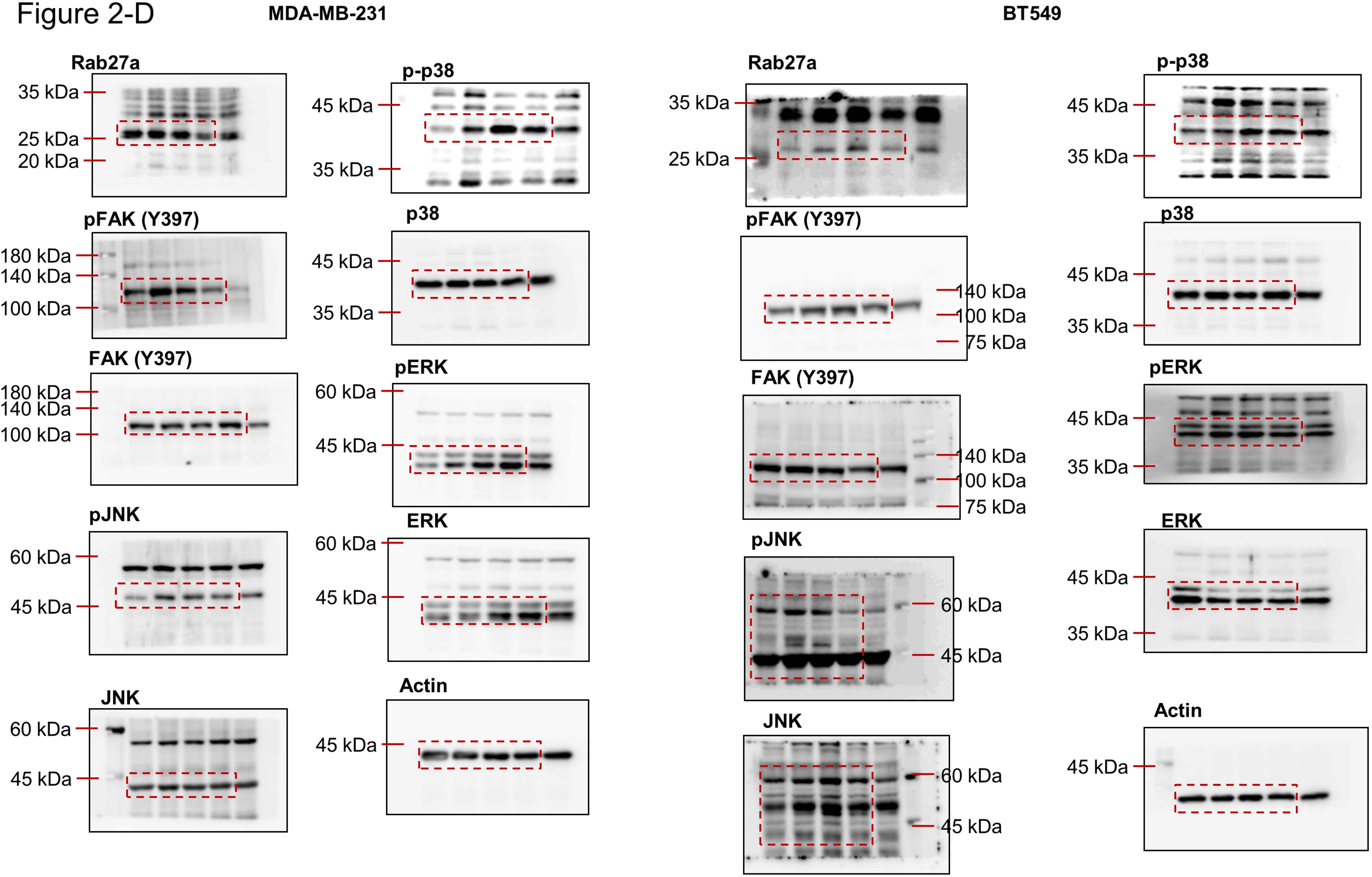
MDA-MB-231
BT549
Rab27a
p-p38
pFAK (Y397)
p38
FAK (Y397)
pERK
pJNK
ERK
JNK
Actin
180 kDa
140 kDa
100 kDa
75 kDa
60 kDa
45 kDa
35 kDa
25 kDa
20 kDa

Figure 3-B

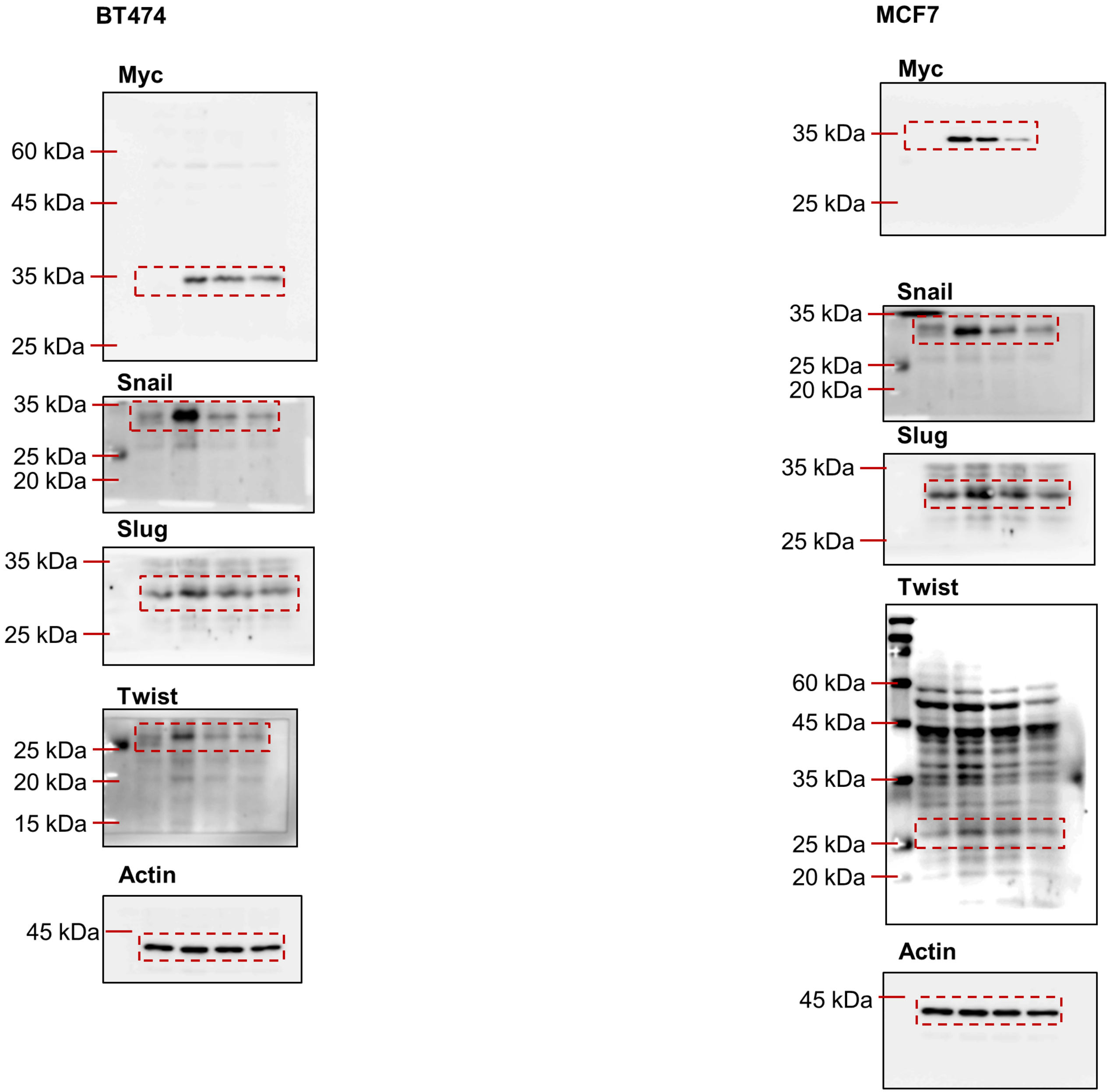

BT474
Myc
60 kDa
45 kDa
35 kDa
25 kDa
Snail
35 kDa
25 kDa
20 kDa
Slug
35 kDa
25 kDa
Twist
25 kDa
20 kDa
15 kDa
Actin
45 kDa
MCF7
Myc
35 kDa
25 kDa
Snail
35 kDa
25 kDa
20 kDa
Slug
35 kDa
25 kDa
Twist
60 kDa
45 kDa
35 kDa
25 kDa
20 kDa
Actin
45 kDa

Figure 3-C

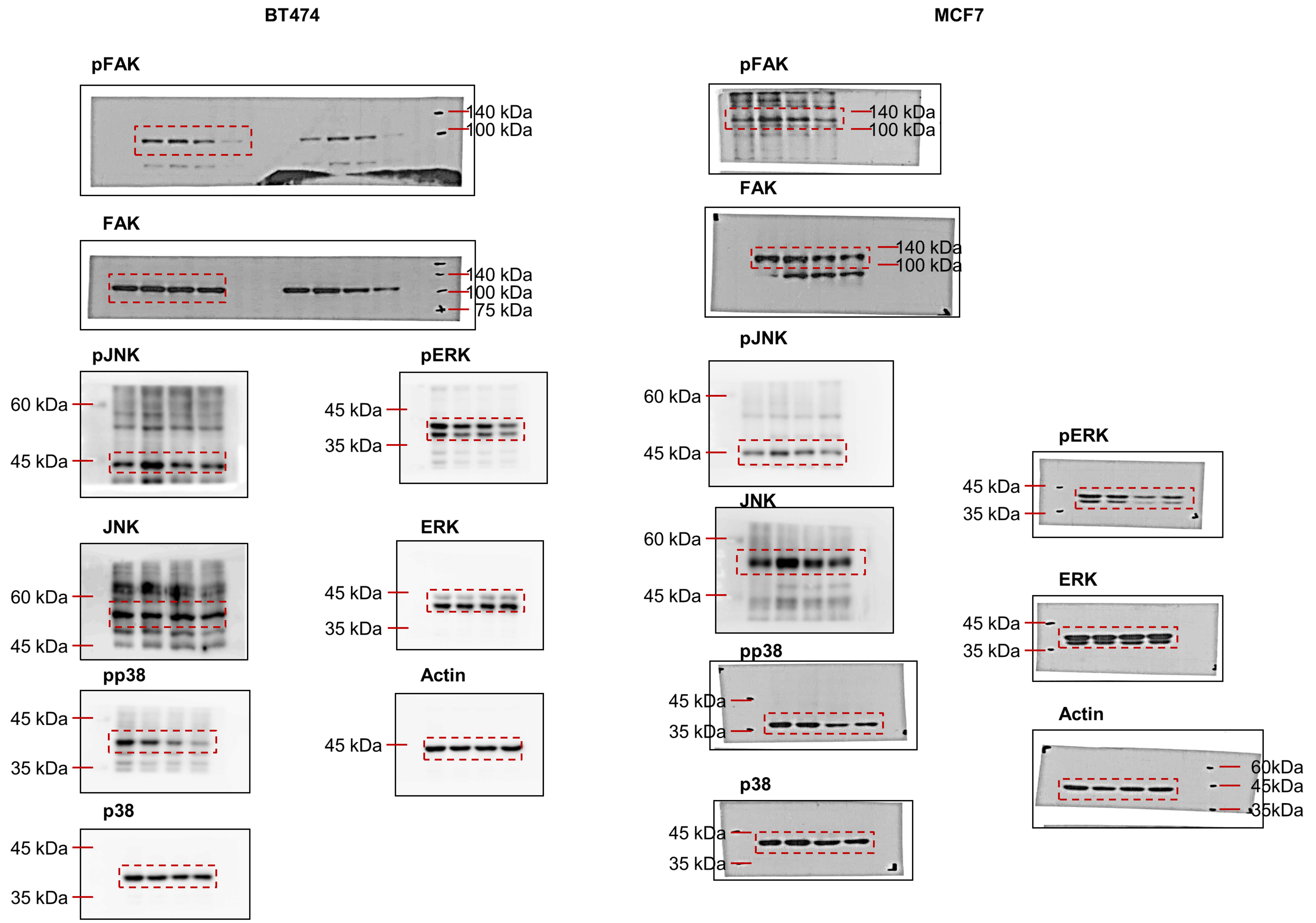

BT474
MCF7
pFAK
140 kDa
100 kDa
FAK
140 kDa
100 kDa
75 kDa
pJNK
60 kDa
45 kDa
pERK
45 kDa
35 kDa
JNK
60 kDa
45 kDa
ERK
45 kDa
35 kDa
pp38
45 kDa
35 kDa
Actin
45 kDa
p38
45 kDa
35 kDa
pFAK
140 kDa
100 kDa
FAK
140 kDa
100 kDa
pJNK
60 kDa
45 kDa
JNK
60 kDa
45 kDa
pp38
45 kDa
35 kDa
p38
45 kDa
35 kDa
pERK
45 kDa
35 kDa
ERK
45 kDa
35 kDa
Actin
60kDa
45kDa
35kDa

Figure 3-D

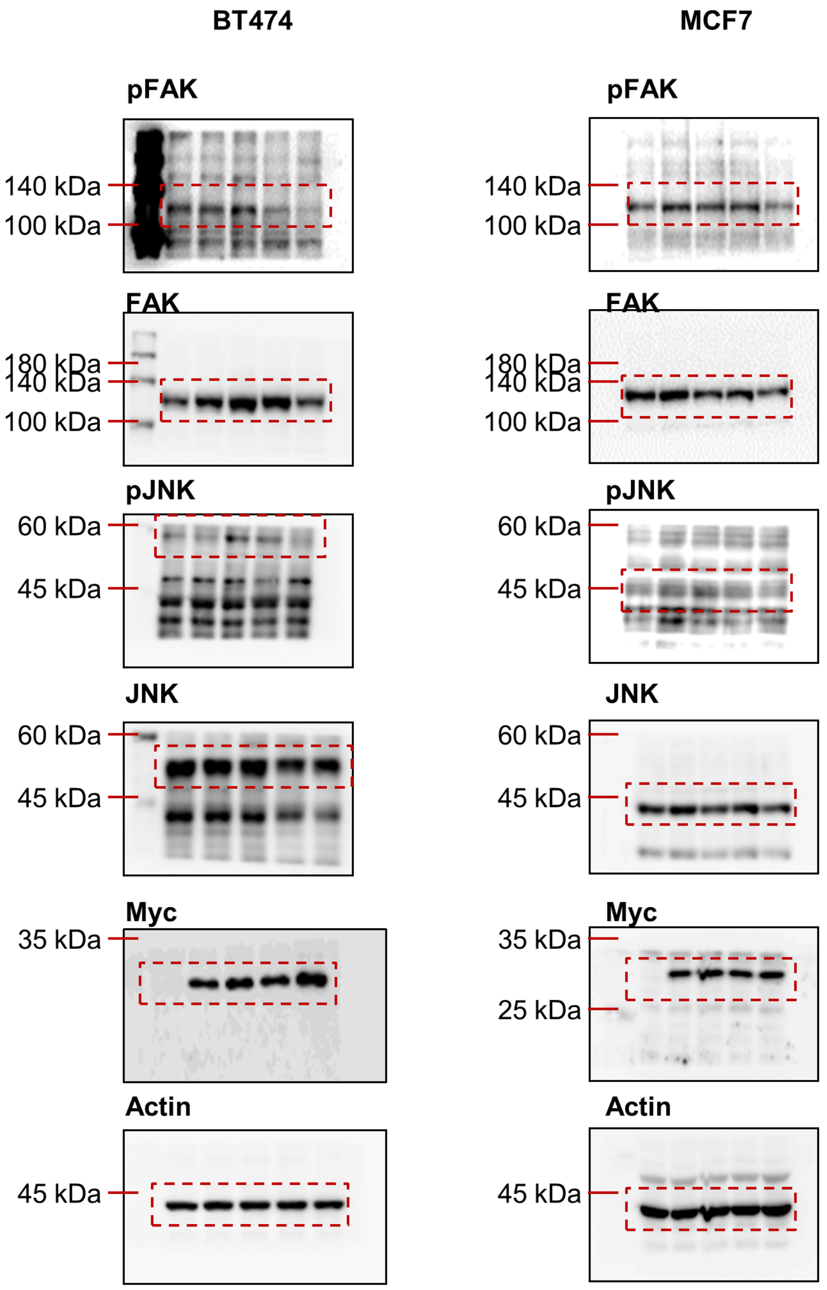

Figure 3-E

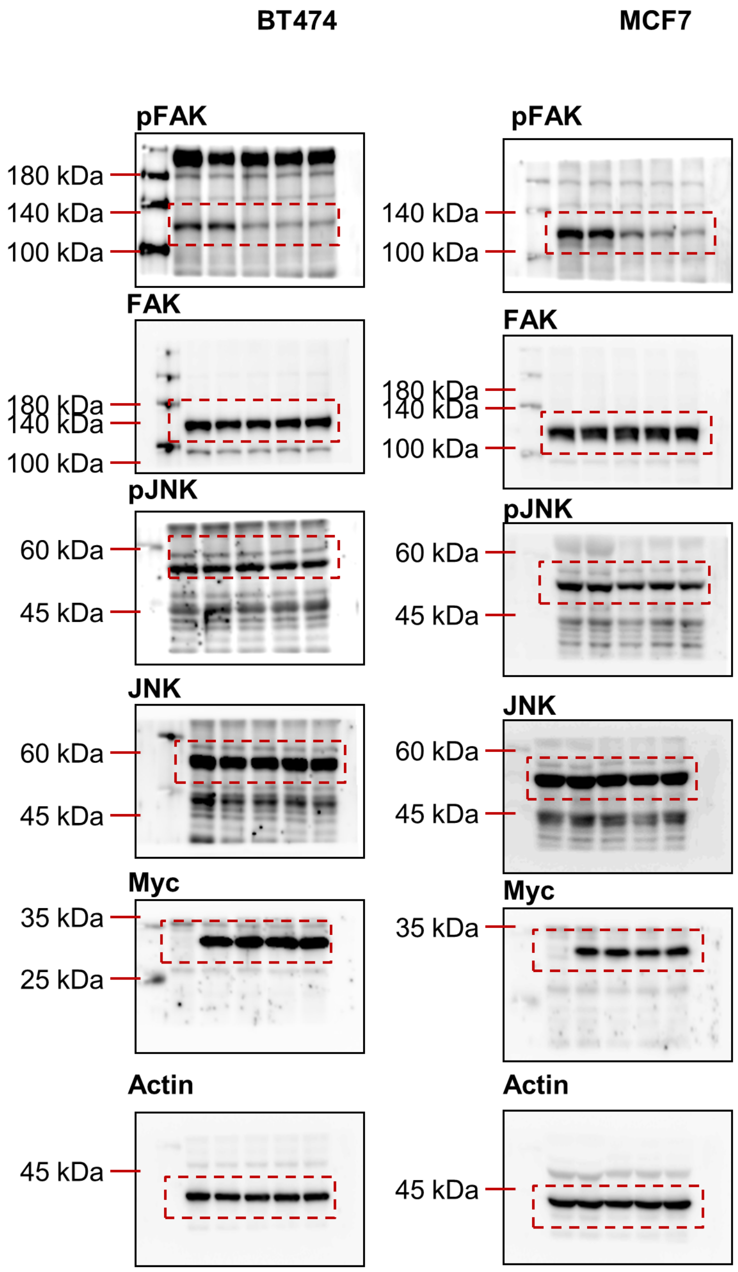

Figure 4-A

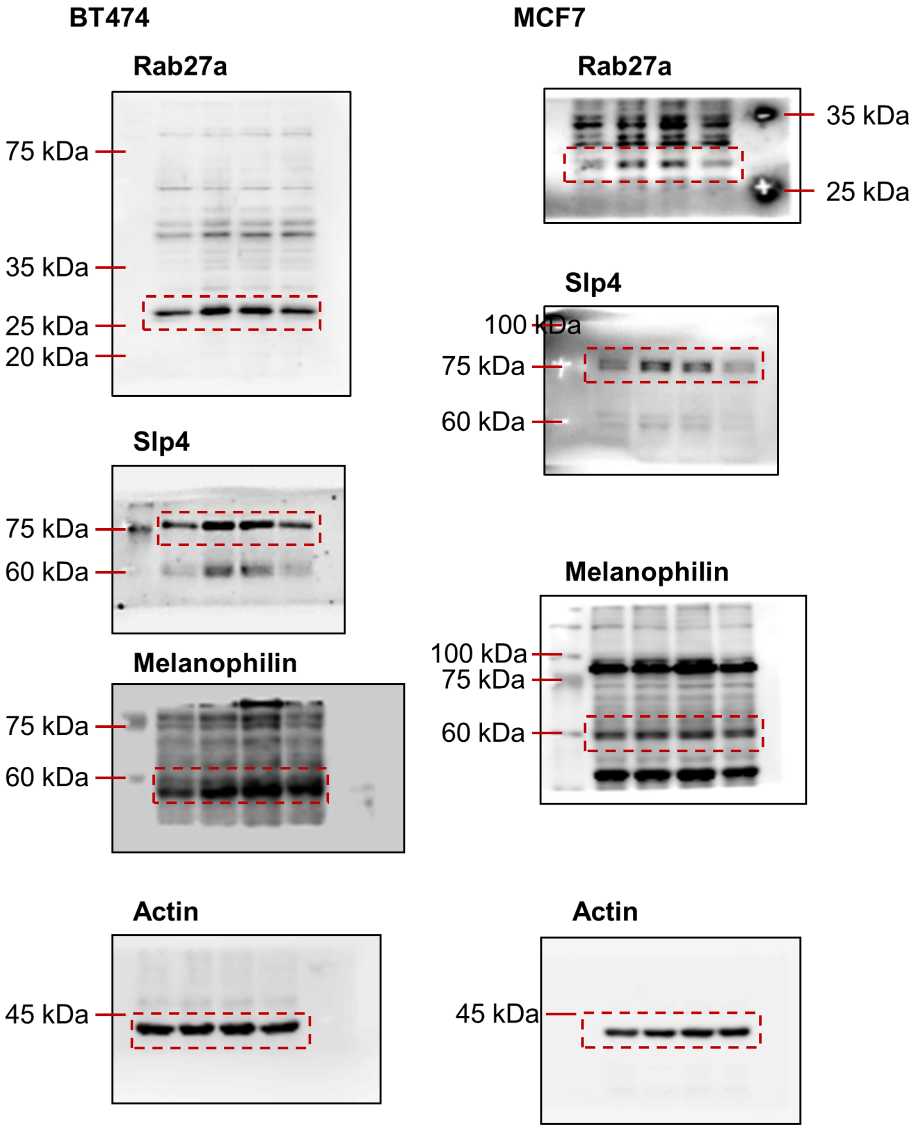

Figure 4-D

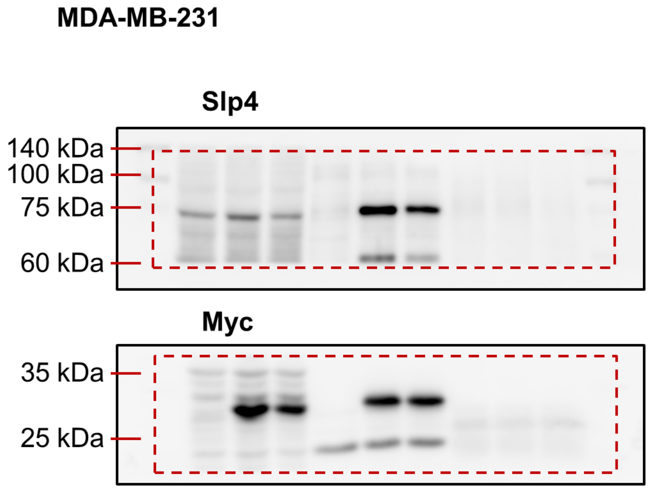

Figure 5-B

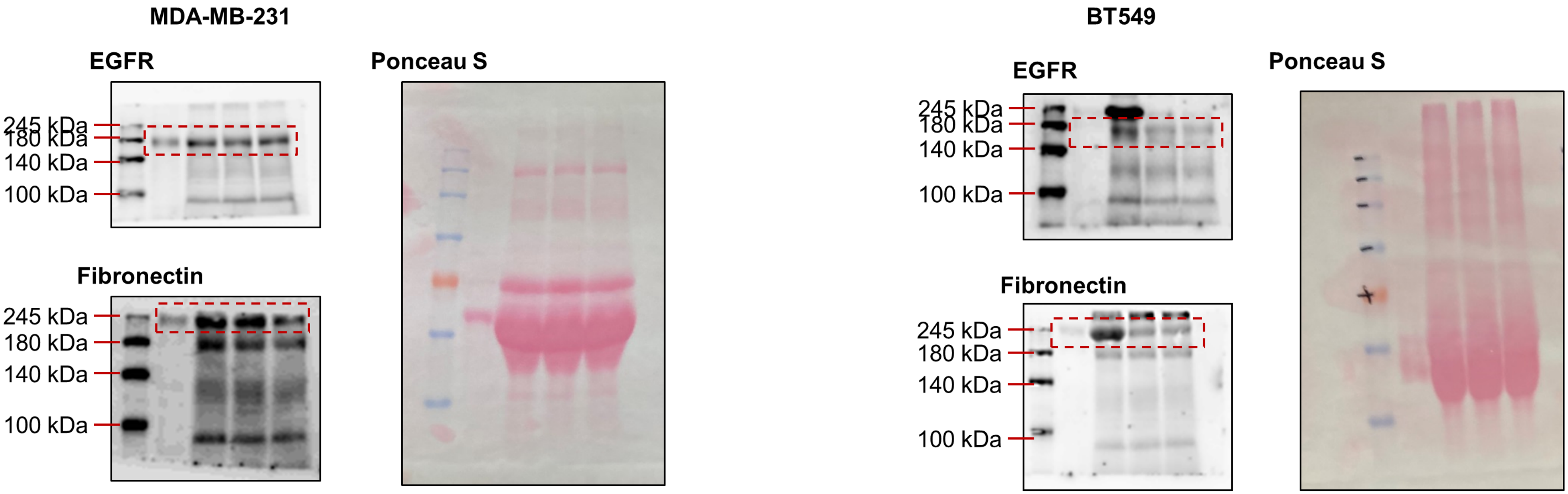
MDA-MB-231
EGFR
245 kDa
180 kDa
140 kDa
100 kDa
Fibronectin
245 kDa
180 kDa
140 kDa
100 kDa
Ponceau S
BT549
EGFR
245 kDa
180 kDa
140 kDa
100 kDa
Fibronectin
245 kDa
180 kDa
140 kDa
100 kDa
Ponceau S
